# A Novel Autonomous Celestial Integrated Navigation for Deep Space Exploration Based on Angle and Stellar Spectra Shift Velocity Measurement

**DOI:** 10.3390/s19112555

**Published:** 2019-06-04

**Authors:** Xiao Chen, Zhaowei Sun, Wei Zhang, Jun Xu

**Affiliations:** 1 Harbin Institute of Technology, Harbin 150001, China; sunzhaowei@hit.edu.cn; 2 Shanghai Institute of Satellite Engineering, Shanghai 201109, China; deepspace509@126.com (W.Z.); junxu509@163.com (J.X.); 3 Shanghai Key Laboratory of Deep Space Exploration Technology, Shanghai 201109, China

**Keywords:** autonomous navigation, integrated navigation, stellar spectra shift velocity measurement

## Abstract

Traditional autonomous celestial navigation usually uses astronomical angle as measurement, which is a function of spacecraft’s position and can’t resolve the spacecraft’s velocity directly. To solve this problem, velocity measurement by stellar spectra shift is proposed in this paper. The autonomous celestial integrated navigation method is derived by combining velocity measurement with angle measurement, which can ensure the long-term high accuracy, real-time and continuous navigation performance for deep space exploration (DSE) missions. The observability of the integrated navigation system is analyzed. Moreover, the design of doppler navigator and hardware in-the-loop simulation system are described. Finally, a simulation example is employed to demonstration the feasibility and effectiveness of the proposed navigation algorithm.

## 1. Introduction

Compared with near-Earth missions, higher navigation performance is required by deep space exploration (DSE) missions for the complicated environment such as long flight distance, many unknown factors of the environment, complicated flight procedures, high communication delay and loss, tracking blind and celestial shelter, etc. Autonomous celestial navigation is a key technology that determines the success of the deep space exploration missions [1]. 

Recently, DSE missions implemented aboard most of the deep space probes possess partial autonomous navigation ability. In addition, part of the landing patrol robot also has the function of autonomous navigation and control [2,3]. The autonomous navigation method has become an effective supplementary method of ground measure [4]. There are many limitations in ground radio navigation such as real-time performance, operation cost and satellite resources, etc. Fortunately, autonomous navigation in DSE can not only overcome these limitations, but also improve the independent survival ability of a deep space probe [5]. Besides, if the accuracy and continuity of DSE navigation can be enhanced, the reliability of mission success can be enhanced as well. In some special flight phases, such as closing, fly-around, landing, adhesion and increased rendezvous, the precise position and velocity information relative to the target objects are required, and the autonomous navigation and control method can perform better than ground measure in these DSE mission [6,7,8].

The traditional autonomous navigation methods mostly depend on angle measurement or ranging information to obtain the real-time estimation state of probe currently [9]. However, it is difficult to obtain the long-term real-time and continuous navigation information because of the limitation of the target object condition [10,11,12]. The astronomical optical information contains a high amount data of spectral characteristics and frequency shift, and the velocity of the probe can be resolved by these information [13]. Once the space and natural resources can be utilized, such as the frequency shift of the characteristic spectral lines in the visible spectral band of stars, the velocity information of spacecraft can be obtained. It will be greatly advantageous to realize the high precision estimation of instantaneous velocity in DSE, and the autonomous navigation accuracy of DSE can be improved further as a result [14,15,16,17].

In this paper, a practical guide is proposed to develop and realize an autonomous celestial navigation based on the spectrum velocity measurement technology in DSE, which can improve the velocity estimation accuracy of angle navigation and inhibit the divergence of position estimation of velocity navigation as well.

The remaining parts of the paper are organized as follows: The principle of integrated navigation is presented in Section 2, followed by the integrated navigation system model in Section 3. Section 4 presents the filtering algorithm. The observability analysis and the design of doppler navigator and hardware in-the-loop simulation system are given in Section 5 and Section 6, respectively. A comparison simulation example of navigation performance between the traditional angle navigation and the integrated navigation based on velocity measurement by stellar spectra shift is performed in Section 7. Finally, conclusions are summarized in Section 8.

## 2. Principle of Integrated Navigation

### 2.1. Velocity Measurement Scheme

Supposing the probe position relative to the stars is static when the probe is receiving the spectrum information of the star, the astronomical optical information is constant, and the wavelength of the stellar spectrum is denoted by $\lambda_{rest}$. When the relative position of the deep space probe to the star has been changed, the wavelengths drift can be detected by the space borne spectrometer, which is denoted by Δλ. The wavelength drift performance on the spectrum is the movement of the spectral line [18,19]. According to the principle of Doppler, the ratio of the wavelength drift value and the wavelength value is equal to the ratio of the line of sight velocity $v_{r}$ and the speed of light c, namely

(1) $$\frac{\Delta \lambda }{\lambda_{rest}}=\frac{v_{r}}{c}.$$

Star spectrometer can obtain the information of the spectral frequency shift phase Δφ, so the relative velocity of the probe and star is resolved indirectly

(2) $$v_{Spe}=\frac{c\cdot \Delta \varphi }{4\pi \cdot \Delta d\cdot \sigma },$$

where Δd is the optical path difference, which is a constant; and σ is the light wave number which is the reciprocal of wavelength of light.

The relative velocity derived from the astronomical spectra Doppler information is scalar value. Supposing the velocity of probe and stars in the heliocentric ecliptic inertial coordinate system as v and $v_{Star}$, and the relative line of sight vector of probe and star is $l_{Star}$, the relative velocity value of probe and star is

(3) $${\left(v_{Star}-v\right)}^{T}l_{Star}=v_{Spe},$$

where $v_{Star}$ and $l_{Star}$ is the velocity and line of sight vector of the star in the inertial coordinate system, respectively. Because the probe position value is much smaller than the star position value, the influence of the probe position is neglected. The value of $v_{Star}$ and $l_{Star}$ can be derived by the star catalogue, such as the Hipparcos and Tycho Star catalogues.

**Figure 1:**
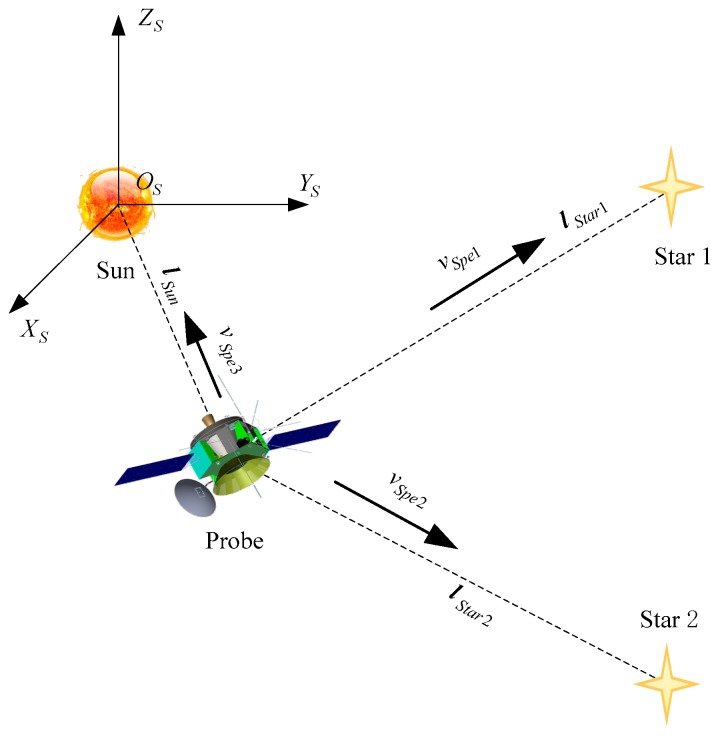
Principle of velocity measurement navigation.

Based on the above analysis, the measurement strategy including the relative velocity measurement of the probe and the sun, and the relative velocity measurement of the probe and the other two stars can be established, which is shown in Figure 1. The equation of the velocity measurement can be written as

(4) $$\left[\begin{array}{c} l_{Star1}^{T}\\ l_{Star2}^{T}\\ l_{Sun}^{T} \end{array}\right]v=\left[\begin{array}{c} l_{Star1}^{T}v_{Star1}\\ l_{Star2}^{T}v_{Star2}\\ l_{Sun}^{T}v_{sun} \end{array}\right]-\left[\begin{array}{c} v_{Spe1}\\ v_{Spe2}\\ v_{Spe3} \end{array}\right].$$

The equation above is linear equation relative to v, so as long as the coefficient matrix $A=\left[\begin{array}{ccc} l_{Star1}^{T} & l_{Star2}^{T} & l_{Sun}^{T} \end{array}\right]$ is invertible, which means the lines of sight vectors of probe and the three stars are non-coplanar, the velocity of the probe in the inertial reference system can be derived directly by the measurement strategy.

### 2.2. Angle Measurement Scheme

**Figure 2:**
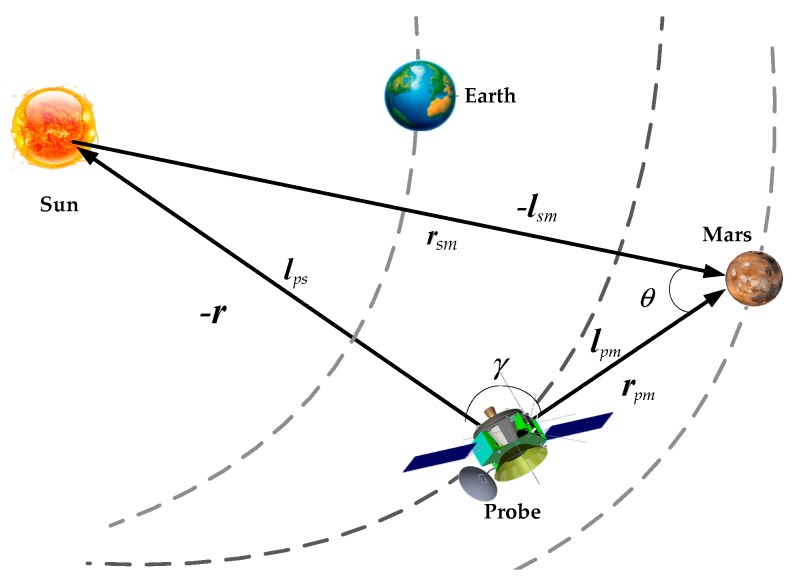
Principle of angle measurement navigation.

With the line of sight vector of sun included in the scheme above, then introducing a close planet position vector, the probe position information can be directly resolved [20,21,22]. The principle of the angle measurement scheme is shown in Figure 2.

In the heliocentric ecliptic inertial coordinate system, according to the geometrical relation of each vector as shown in Figure 2, there are equations below

(5) $$\begin{array}{l} \gamma =\arccos\left(l_{ps}\cdot l_{pm}\right)\\ \theta =\arccos\left(l_{sm}\cdot l_{pm}\right) \end{array}$$

where $l_{ps}$ is the relative line of sight vector of probe and star, $l_{pm}$ is the relative line of sight vector from probe to planet, such as Mars, and $l_{sm}$ is the relative line of sight vector from sun to planet.

The equation about the position of probe ***r*** can be written as

(6) $$\begin{array}{ll} r & =-\frac{\left\|r_{sm}\right\|\sin\theta }{\sin\gamma }\cdot l_{ps}\\ & =-\left\|r_{sm}\right\|\sqrt{\frac{1-{\left(\cos\theta \right)}^{2}}{1-{\left(\cos\gamma \right)}^{2}}}\cdot l_{ps} \end{array}$$

Equation (6) can be rewritten as

(7) $$r=-\left\|r_{sm}\right\|\sqrt{\frac{1-{\left(l_{sm}\cdot l_{pm}\right)}^{2}}{1-{\left(l_{ps}\cdot l_{pm}\right)}^{2}}}\cdot l_{ps},$$

where $r_{sm}$ is the position of Mars and the position of probe can be resolved by the equation above.

In summary, the autonomous integrated navigation system is derived by combining the velocity measurement with the angle measurement. The principle of the navigation system is clear and easy to realize.

## 3. Integrated Navigation System Model

### 3.1. State Model

The state model of DSE navigation is generally established by the orbit dynamics. In the cruise phase of the Mars exploration mission, gravitational perturbation of sun and planets, sunlight pressure perturbation, and probe thrust have been considered in the model [23,24]. Supposing the probe position vector is $r={\left[x,y,z\right]}^{T}$ and the velocity vector is $v={\left[v_{x},v_{y},v_{z}\right]}^{T}$, the state vector is $X\left(t\right)={\left[x,y,z,v_{x},v_{y},v_{z}\right]}^{T}$, so the state model can be written as

(8) $$\overset{\cdot }{r}=v,$$

(9) $$\overset{\cdot \cdot }{r}=-\frac{\mu_{s}}{r^{3}}r+\sum_{i=1}^{N}\mu_{i}\left[-\frac{r_{i}}{r_{i}^{3}}+\frac{r_{si}}{r_{si}^{3}}\right]+\eta P_{SR}AU^{2}C_{R}\left(\frac{A_{R}}{m}\right)\frac{r}{r^{3}}+a_{T},$$

where the first term is the gravitational perturbation of sun, and $\mu_{S}$ is the gravitational constant. The second part refers to the gravitational perturbations of planets, where $\mu_{i}$ is the relevant gravitational constant, i=1,2,3,… is the number of planet, $r_{si}$ represents the relative position from probe to planet. The third part is sunlight pressure perturbation, where η is the shading factor, and $P_{SR}$ is the value of sunlight pressure perturbation in 1AU, $C_{R}$ is the surface reflection coefficient of probe, $A_{R}$ is the cross-sectional area of probe in the direction perpendicular to the sun’s rays, and m is the mass of probe. The last part is probe thrust.

Commonly, the equation of the state model above can be expressed as the following general form

(10) $$\overset{\cdot }{X(t)}=f(X(t),t)+W(t),$$

where W(t) is the state noise.

### 3.2. Measurement Model

As described in Section 2, assuming $Z_{v}(t)={\left[v_{Spe1},v_{Spe2},v_{Spe3}\right]}^{T}$, the velocity measurement equation can be written as

(11) $$Z_{v}(t)=h_{v}(X(t),t)+V_{v}(t),$$

where

(12) $$h_{v}(X(t),t)=\left[\begin{array}{c} l_{Star1}^{T}\cdot \left(v_{Star1}-v\right)\\ l_{Star2}^{T}\cdot \left(v_{Star2}-v\right)\\ \frac{v\cdot r}{r} \end{array}\right]$$

and $V_{v}(t)$ is the velocity measurement noise.

Assuming $Z_{l}(t)={\left[l_{ps},l_{pm}\right]}^{T}$, the angle measurement equation can be written as

(13) $$Z_{l}(t)=h_{l}(X(t),t)+V_{l}(t),$$

where

(14) $$h_{l}(X(t),t)=\left[\begin{array}{c} l_{ps}\\ l_{pm} \end{array}\right]$$

and $V_{l}(t)$ is the angle measurement noise. 

## 4. Filtering Algorithm

Unscented Kalman Filter (UKF) is suitable for the nonlinear system [25]. The unscented transformation (UT) is the core of the UKF filtering algorithm [26]. The UT method selects a set of sample points nearby $\hat{x}\left(k|k\right)$, and the mean and the covariance of these sample points respectively are $\hat{x}\left(k|k\right)$ and P(k|k). Assuming the state variables are n×1, the 2n+1 sample points and their weights are

(15) $$\chi_{0,k}={\hat{x}}_{k},$$

(16) $$\begin{array}{l} \chi_{i,k}={\hat{x}}_{k}+\sqrt{n+\lambda }{\left(\sqrt{P(k|k)}\right)}_{i}\left(i=1,2,\ldots ,n\right)\\ \chi_{i+n,k}={\hat{x}}_{k}-\sqrt{n+\lambda }{\left(\sqrt{P(k|k)}\right)}_{i}\left(i=1,2,\ldots ,n\right) \end{array}$$

(17) $$\begin{array}{l} w_{0}^{m}=\frac{\lambda }{\left(n+\lambda \right)}\\ w_{0}^{c}=\frac{\lambda }{\left(n+\lambda \right)}+1-\alpha^{2}+\beta \\ w_{i}^{m}=w_{i}^{c}=\frac{1}{2(n+\lambda )}(i=1,2,\ldots ,2n) \end{array}$$

where

$$\lambda =\alpha^{2}(n+\kappa )-n,$$

where α=1, κ=3−n, β=2.

The standard UKF algorithm can be described as follows.

(1) Initialization:

(18) $$\begin{array}{l} {\hat{x}}_{0}=E[x_{0}]\\ P_{0}=E[(x_{0}-{\hat{x}}_{0}){\left(x_{0}-{\hat{x}}_{0}\right)}^{T}]. \end{array}$$

(2) Computation sample points:

(19) $$\chi_{k-1}=\left[\begin{array}{ccc} {\hat{x}}_{k-1} & {\hat{x}}_{k-1}+\sqrt{\left(n+\lambda \right)P_{k-1}} & {\hat{x}}_{k-1}-\sqrt{\left(n+\lambda \right)P_{k-1}} \end{array}\right].$$

(3) Time update:

(20) $$\chi_{k|k-1}=f\left(\chi_{k-1},k-1\right),$$

(21) $${\hat{x}}_{k}^{-}=\sum_{i=0}^{2n}w_{i}\chi_{i,k|k-1},$$

(22) $$P_{k}^{-}=\sum_{i=0}^{2n}w_{i}\left[\chi_{i,k|k-1}-{\hat{x}}_{k}^{-}\right]{\left[\chi_{i,k|k-1}-{\hat{x}}_{k}^{-}\right]}^{T}+Q_{k-1},$$

(23) $$Z_{k|k-1}=h(\chi_{k|k-1},k),$$

(24) $${\hat{z}}_{k}^{-}=\sum_{i=0}^{2n}w_{i}Z_{i,\;k|k-1}.$$

(4) Measurement update:

(25) $$P_{{\hat{z}}_{k}{\hat{z}}_{k}}=\sum_{i=0}^{2n}w_{i}\left[Z_{i,k|k-1}-{\hat{z}}_{k}^{-}\right]{\left[Z_{i,k|k-1}-{\hat{z}}_{k}^{-}\right]}^{T}+R_{k},$$

(26) $$P_{{\hat{x}}_{k}{\hat{z}}_{k}}=\sum_{i=0}^{2n}w_{i}\left[\chi_{i,k|k-1}-{\hat{x}}_{k}^{-}\right]{\left[Z_{i,k|k-1}-{\hat{z}}_{k}^{-}\right]}^{T},$$

(27) $$K_{k}=P_{{\hat{x}}_{k}{\hat{z}}_{k}}P_{{\hat{z}}_{k}{\hat{z}}_{k}}^{-1},$$

(28) $${\hat{x}}_{k}={\hat{x}}_{k}^{-}+K_{k}\left(Z_{k}-{\hat{z}}_{k}^{-}\right),$$

(29) $$P_{k}=P_{k}^{-}-K_{k}P_{{\hat{z}}_{k}{\hat{z}}_{k}}K_{k}^{T},$$

where $Q_{k-1}$ and $R_{k}$ is respectively refer to the system and measurement noise covariance matrix.

## 5. Observability of Integrated Navigation System

The observability of the navigation system reflects the ability of the system to determine the state of the system through observation and measurement in limited time [27]. The observable matrix is an important basis for observable analysis of the system. In view of the strong nonlinearity of deep space exploration autonomous navigation systems, the Lie derivation can be used.

Ignoring the influence of errors, the navigation system equation can be generally expressed as

(30) $$\sum :\{\begin{array}{c} \overset{\cdot }{X}=f\left(X\right)\\ Z=h\left(X\right) \end{array},$$

where state vector $X\in X^{n}\subset R^{n}$, and equation of state f and equation of observation h are smooth analytic functions in $C^{n}$.

According to differential geometry theory, the *k*th order Lie derivation of the nonlinear navigation system is [28]

(31) $$L_{f}^{0}h\left(X\right)=h\left(X\right),\;k=0,$$

(32) $$L_{f}^{k}h\left(X\right)=\frac{\partial \left(L_{f}^{k-1}h\right)}{\partial X}f\left(X\right),\;k=1,2,\ldots$$

where H can be derived by the equations above for the nonlinear navigation system. So $H^{n}=\left\{h,L_{f}h,\ldots ,L_{f}^{n-1}h\right\}$ is the minimum linear space which contains the state and measurement variables. When $X_{0}\in X^{n}$, if $dH^{n}$ match the observable rank condition, ∑ is partial weak observable for $X_{0}$ [29,30].

The observable matrix Q(X) can be defined by $dH^{n}$ as

(33) $$Q\left(X\right)=\left[\begin{array}{c} dL_{f}^{0}h\left(X\right)\\ dL_{f}^{1}h\left(X\right)\\ \vdots \\ dL_{f}^{n-1}h\left(X\right) \end{array}\right].$$

where

(34) $$dL_{f}^{k}h\left(X\right)=\frac{\partial \left(L_{f}^{k}h\right)}{\partial X},\;k=0,1,2,\ldots$$

The observable degree can be derived by condition number of the observability matrix above, which means

(35) $$\gamma =\frac{1}{cond\left(Q\right)}=\frac{1}{\left\|Q\right\|\cdot \left\|Q^{-1}\right\|}=\frac{\min\sigma_{Q}}{\max\sigma_{Q}},$$

where $\sigma_{Q}$ is the singular value of Q. Obviously, γ∈[0,1] and when γ=0, which means rank(Q)<n, the system is unobservable.

## 6. Doppler Navigator and Hardware In-The-Loop Simulation System

### 6.1. Design of Doppler Navigator Based On Atomic Frequency Discrimination

**Figure 3:**
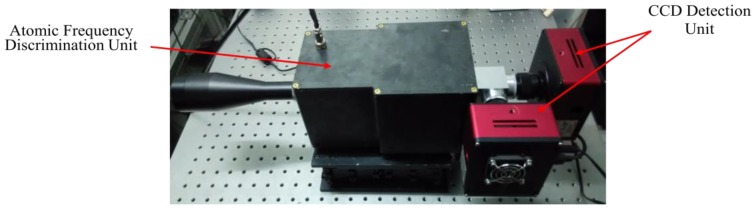
The components of navigator.

The navigator is composed of an atomic frequency discrimination unit, a Charge Coupled Device (CCD) detection unit and a speed measuring computer unit, as shown in Figure 3.

The atomic frequency discrimination unit consists of two main components: receiving filter, and atomic frequency discrimination. The receiving filter module is composed of receiving telescope and pre-filter, which mainly realizes the function of receiving sunlight and filtering ultraviolet and infrared light. The atomic frequency discrimination module is composed of a two-peak atomic frequency discriminator and a peak-selecting atomic frequency discriminator, which are used to identify the frequency of solar spectral lines and extract the direction-of-sight velocity information.

The CCD detection unit consists of two CCD detectors, which can image the red shift signal and blue shift signal output by the atomic frequency discrimination unit.

The speed measuring computer unit receives the original image data from the CCD detector, generates the test samples, reduces the error through the algorithm and outputs the speed data.

**Figure 4:**
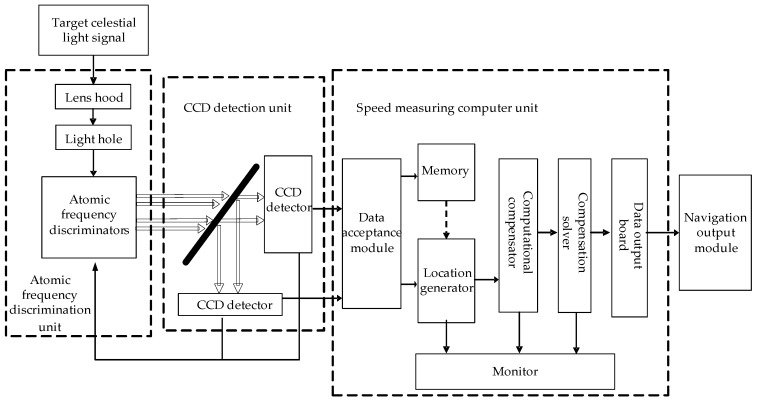
Workflow diagram of navigator.

The workflow of the navigator is shown in Figure 4.

### 6.2. Design of Hardware In-The-Loop Simulation System

dSPACE real-time simulation platform is an ideal tool for a current hardware in-the-loop simulation system [31,32,33,34,35], with a rich and powerful interface. The integrated navigation hardware in-the-loop simulation system consists of the following components: dSPACE real-time simulation platform, angle measurement sensor, velocity measurement sensor, navigation computer, performance evaluation computer, data storage device, Mars image simulator, Sun/star spectrum simulator and the corresponding software.

Considering the universality and flexibility of the hardware in-the-loop simulation system and the portability of the algorithm, the software is designed based on a common development environment and it is convenient for secondary expansion. Hardware in-the-loop simulation system software includes: deep space dynamics simulation software, navigation image simulation software, navigation image acquisition and processing software, Sun/star spectrum simulation software, spectral acquisition and processing software, angle navigation software, velocity navigation software and integrated navigation software.

**Figure 5:**
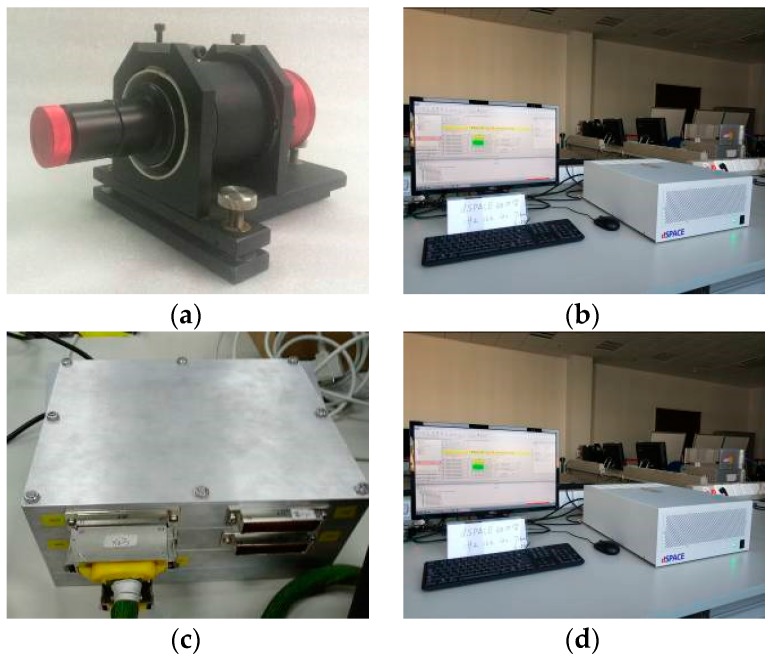
Practicalities of hardware in-the-loop simulation system. (**a**) Mars image simulator; (**b**) dSPACE simulation platform; (**c**) navigation computer; (**d**) spectral shift sensor.

According to the function of the system, the whole system is divided into the following subsystems: deep space dynamic environment simulation subsystem, angle navigation subsystem, velocity navigation subsystem, integrated navigation subsystem and navigation performance evaluation and display subsystem. The practicalities of hardware in-the-loop simulation system is shown as Figure 5.

**Figure 6:**
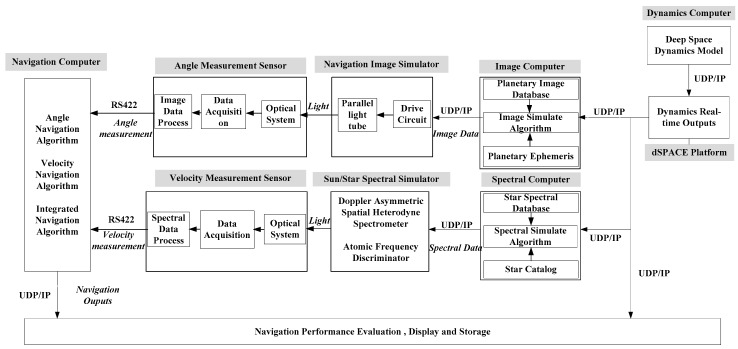
Hardware connection diagram of the simulation system.

According to the functional design of the integrated navigation system, it can be divided into the following components: deep space dynamic environment output module, navigation image output module, navigation image acquisition module, angle navigation information extraction module, Sun/star spectrum output module, velocity measurement navigation information extraction module, integrated navigation filter estimation module, navigation performance evaluation and display module. A hardware connection diagram of the system is shown in Figure 6.

## 7. Simulation and Analysis

Based on the cruise phase of the Mars exploration mission, the integrated navigation method proposed in this paper was analyzed by hardware in-the-loop simulation. The state parameters of real orbit produced by simulation software and the initial parameters of the Mars probe in the heliocentric ecliptic inertial coordinate system are shown in Table 1.

### 7.1. Observability Analysis

According to the observability theory in Section 5, observable results of different navigation methods are shown in Table 2. The observable degree of angle navigation was worse than integrated navigation, which means the navigation performance of integrated navigation was better than angle navigation.

### 7.2. Accuracy Analysis of Intergrated Navigation 

The parameters of the UKF algorithm are shown in Table 3. The influences of the gravitational perturbations of the Sun, Mars and Earth, and the sunlight pressure perturbation were considered in the simulation dynamical model. The planetary information was derived from DE421. The Hipparcos and Tycho star catalogues were adopted to obtain data of the stars, and the catalogue numbers of the two stars in the catalogues are 45348 and 172167.

**Figure 7:**
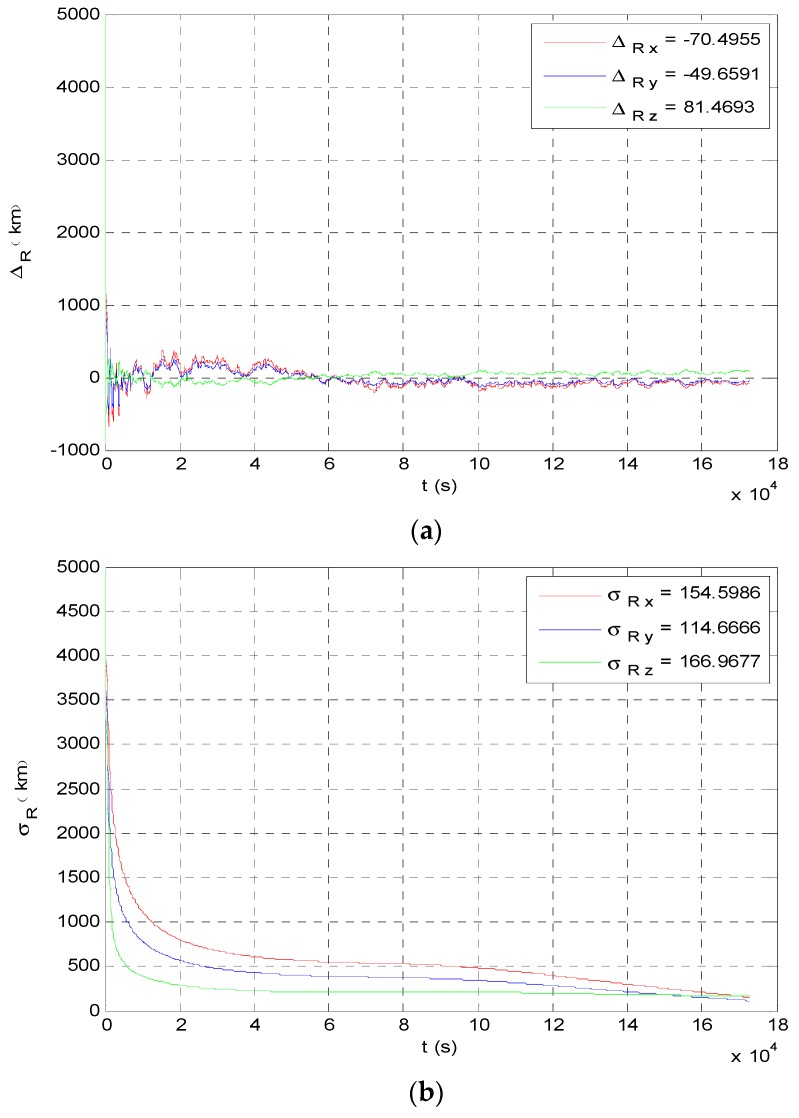
Position estimate results of angle navigation. (**a**) Angle navigation estimation value of probe’s position; (**b**) angle navigation estimation covariance of probe’s position.

**Figure 8:**
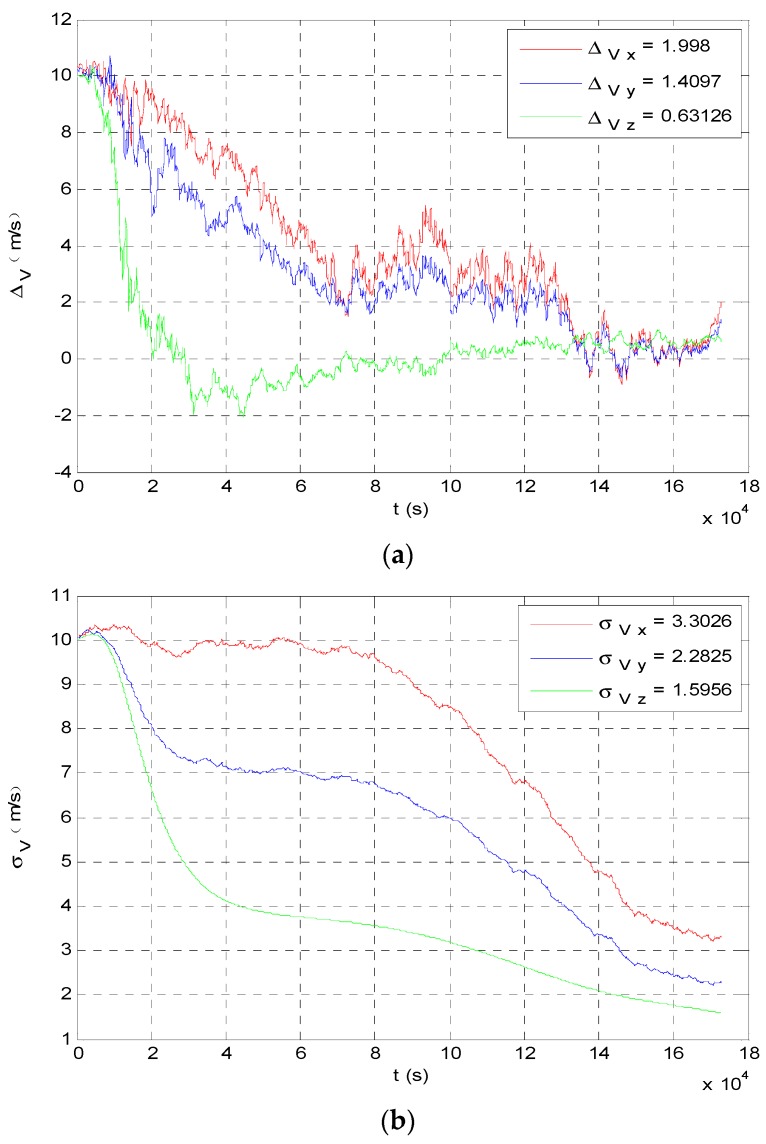
Velocity estimate results of angle navigation. (**a**) Angle navigation estimation value of probe’s velocity; (**b**) angle navigation estimation covariance of probe’s velocity.

**Figure 9:**
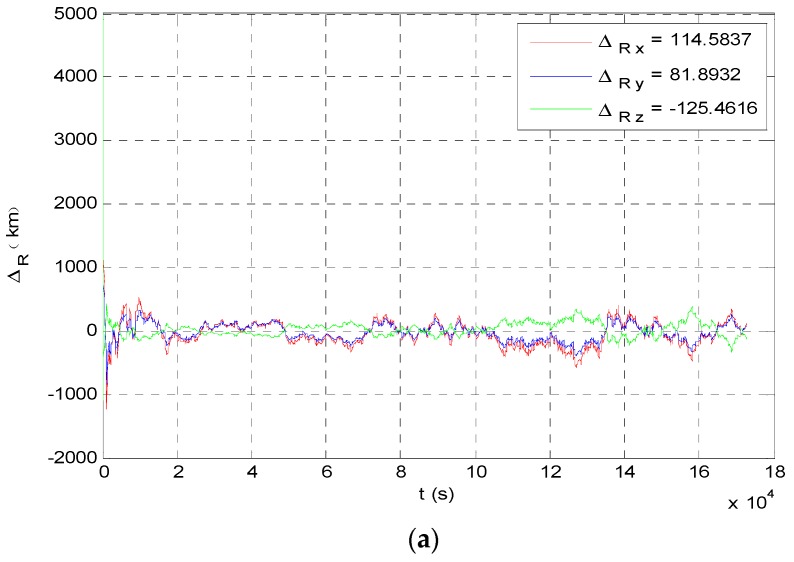

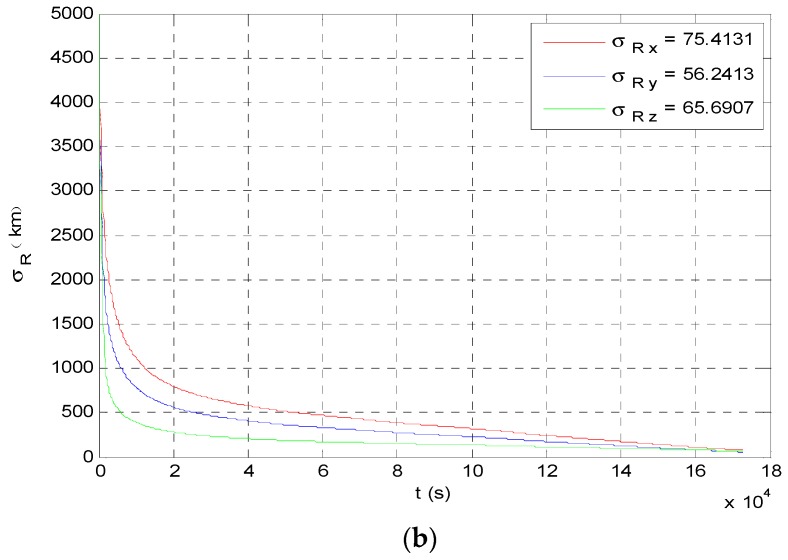
Position estimate results of integrated navigation. (**a**) Integrated navigation estimation value of probe’s position; (**b**) integrated navigation estimation covariance of probe’s position.

**Figure 10:**
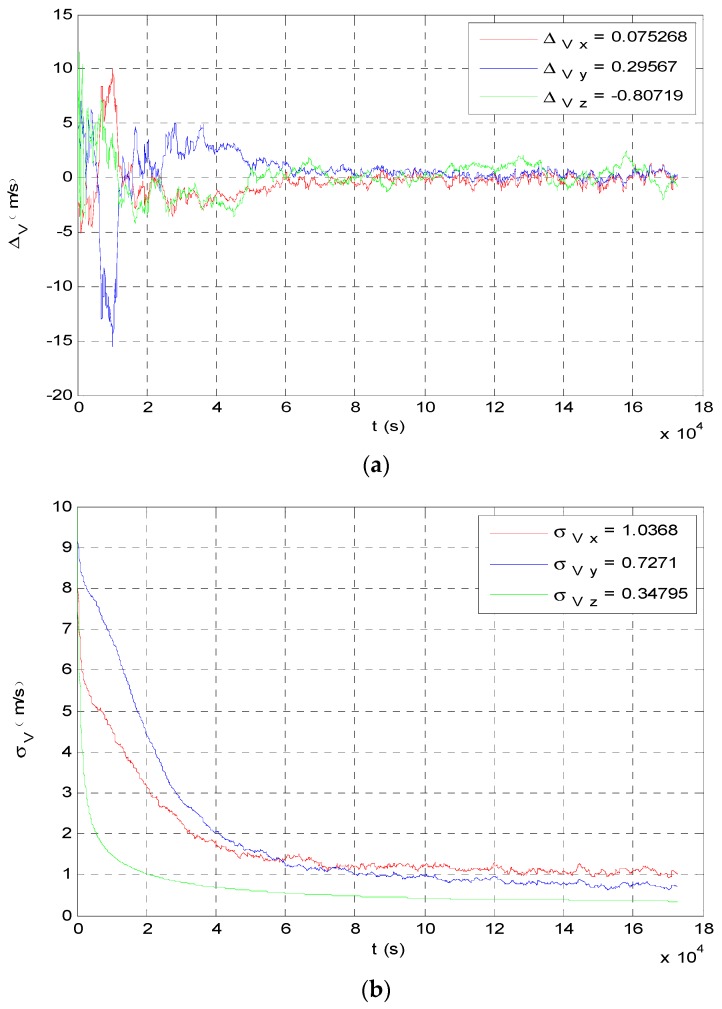
Velocity estimate results of integrated navigation. (**a**) Integrated navigation estimation value of probe’s velocity; (**b**) integrated navigation estimation covariance of probe’s velocity.

The angle navigation simulation results are shown as Figure 7 and Figure 8. The simulation results showed that the accuracy of position estimation was 254.8198 km (3σ), and the accuracy of velocity estimation was 4.3201 m/s (3σ). The integrated navigation simulation results are shown as Figure 9 and Figure 10. The accuracy of position estimation was 114.5353 km (3σ), and the accuracy of velocity estimation was 1.3133 m/s (3σ).

The performance of integrated navigation was better than angle navigation as the introduction of velocity measurement, and the accuracy of velocity was evidently improved. This accorded with the results of observability analysis.

## 8. Conclusions

In this paper, an integrated navigation method based on stellar spectra shift velocity measurement is proposed. The observability of integrated navigation is better than traditional angle navigation. The simulation results demonstrate that the proposed method has a better performance than traditional celestial angle navigation. This research is a practical guide to the development and realization of the autonomous integrated celestial navigation based on spectrum velocity measurement technology in DSE. Furthermore, the integrated navigation system can combine other navigation methods according to different stages of deep space exploration, such as combining radio navigation, X-ray pulsar navigation during interstellar cruise, or autonomous navigation based on sequential images in the landing stage.

## Figures and Tables

**Table 1:** The basic orbit parameters.

Symbol	Quantity
Initial state	**r** = [60480784, 216398917, 6349369] km **v** = [ −20.2006, 10.0324, −0.4970] km/s
Initial bias	**Δr** = [5000, 5000, 5000] km **Δv** = [100, 100, 100] m/s
Initial time	2021-1-15 05:46:07 UTC
End time	2021-1-25 18:40:00 UTC

**Table 2:** Observability analysis results. LOS: Line of Sight; RRV: Relative Radial Velocity.

Navigation Methods	Measurement	Observable Degree
Angle navigation	LOS to Sun and Mars	8.9684 × 10^−11^
Integrated navigation	LOS to Sun and Mars RRV to Sun and other two stars	8.2405 × 10^−8^

**Table 3:** Simulation parameters.

Symbol	Quantity
Step time	600 s
Bias of angle measurement	2 arc sec
Bias of velocity measurement	1 m/s
Unscented Kalman Filter (UKF) basic parameters	$P=\left[\begin{array}{cccccc} 10^{-6} & 0 & 0 & 0 & 0 & 0\\ 0 & 10^{-6} & 0 & 0 & 0 & 0\\ 0 & 0 & 10^{-6} & 0 & 0 & 0\\ 0 & 0 & 0 & 10^{-2} & 0 & 0\\ 0 & 0 & 0 & 0 & 10^{-2} & 0\\ 0 & 0 & 0 & 0 & 0 & 10^{-2} \end{array}\right]$ $Q=10^{-20}I_{6\times 6}$, $R=0.235\times 10^{-10}I_{6\times 6}$ α=1, β=2, κ=−3

## References

[1] Fang J.C., Ning X.L., Liu J. (2017). Principles and Methods of Spacecraft Celestial Navigation.

[2] Zhang S., Dong Y.T., Ouyang Y.C., Yin Z., Peng K.X. (2018). Adaptive Neural Control for Robotic Manipulators with Output Constraints and Uncertainties. IEEE Trans. Neutr. Netw. Learn. Syst..

[3] He W., Dong Y.T. (2018). Adaptive Fuzzy Neural Network Control for a Constrained Robot Using Impedence Learning. IEEE Trans. Neutr. Netw. Learn. Syst..

[4] Ning X.L., Fang J.C. (2009). A new autonomous celestial navigation method for the lunar rover. Robot. Auton. Syst..

[5] Zhang W., Chen X., You W., Fang B. (2013). New autonomous navigation method based on red shift. Aerosp. Shanghai.

[6] Huang Q.L., Chen X., You W. Error analysis of the autonomous celestial navigation based on the spectrum velocity measurement. Proceedings of the 6th CSA/IAA Conference on Advanced Space Technology.

[7] Wang W., Fang B.D., Zhang W. Deceleration options for a robotic interstellar spacecraft. Proceedings of the 64th International Astronautical Congress.

[8] Chen X., Zhang W., Wang W. Preliminary Research of Mars Local Navigation Constellation. Proceedings of the 64th International Astronautical Congress.

[9] Ning X.L., Fang J.C. (2007). An autonomous celestial navigation method for LEO satellite based on unscented Kalman filter and information fusion. Aerosp. Sci. Technol..

[10] Wei E.H., Yang H.Z., Zhang S., Liu J.N., Yi H. (2014). Modeling on autonomous navigation of Mars probe with pulsar and non real-time adjustment methods. J. Deep. Space Explor..

[11] Lightsey E.G., Mogensen A., Burkhar P.D., Ely T.A., Duncan C. (2008). Real-Time Navigation for Mars Missions Using the Mars Network. J. Spacecr. Rockets.

[12] Chen X., Huang Q.L. (2015). A novel celestial navigation method for Mars exploration during the interplanetary cruise. Chin. Soc. Opt. Eng. Conf..

[13] Liu J., Fang J.C., Liu G. (2017). Solar frequency shift–based radial velocity difference measurement for formation flight and its integrated navigation. J. Aerosp. Eng..

[14] Cui P.Y., Wang S., Gao A., Yu Z. (2016). X-ray pulsars/Doppler integrated navigation for Mars final approach. Adv. Space Res..

[15] Shearer A., Golden A. (2001). Implications of the optical observations of isolated neutron stars. Astrophys. J..

[16] Yim J.R., Crassidis J., Junkins J. Autonomous orbit navigation of interplanetary spacecraft. Proceedings of the 2000 AIAA/AAS Astrodynamics Specialist Conference.

[17] Liu J., Fang J.C., Ning X.L. (2014). Closed-loop EKF-based Pulsar Navigation for Mars Explorer with Doppler Effects. J. Navig..

[18] Harlander J., Reynolds R.J., Roesler F.L. (1992). Spatial Heterodyne Spectroscopy for the Exploration of Diffuse Interstellar Emission Lines at Far-Ultraviolet Wavelengths. Astrophys. J..

[19] Englert C.R., Babcock D.D., Harlander J.M. (2007). Doppler asymmetric spatial heterodyne spectroscopy (DASH): Concept and experimental demonstration. Appl. Opt..

[20] Ning X.L., Fang J.C. (2008). Spacecraft autonomous navigation using unscented particle filter-based celestial/Doppler information fusion. Meas. Sci. Technol..

[21] Ma X., Fang J., Ning X., Liu G., Ye H. (2016). A Radio/Optical Integrated Navigation Method Based on Ephemeris Correction for an Interplanetary Probe to approach a Target Planet. J. Navig..

[22] Bo Y. (2016). On-orbit calibration approach for optical navigation camera in deep space exploration. J. Deep Space Explor..

[23] Ma X., Ning X.L., Fang J.C. (2012). Analysis of Orbital Dynamic Equation in Navigation for a Mars Gravity-Assist Mission. J. Navig..

[24] Wang M., Zheng X., Cheng Y., Chen X. (2016). Scheme and Key Technologies of Autonomous Optical Navigation for Mars Exploration in Cruise and Capture Phase. Geomat. Inf. Sci. Wuhan Univ..

[25] Julier S.J., Uhlmann J.K. Unscented Filtering Nonlinear Systems. Proceedings of the American Control Conference.

[26] Qing Y., Zhang H., Wang S. (2012). Kalman Filter and Integrated Navigation Theory.

[27] Huang X., Cui P., Cui H. (2006). Observability Analysis of Deep Space Autonomous Navigation System. J. Astronaut..

[28] Cui P., Chang X., Cui H. (2011). Research on Observability Analysis-Based Autonomous Navigation Method for Deep Space. J. Astronaut..

[29] Hermann R., Krener A.J. (1977). Nonlinear controllability and observability. IEEE Trans. Auto. Control.

[30] Xin M.A., Chen X., Fang J., Liu G., Ning X. Observability analysis of autonomous navigation for deep space exploration with LOS/TOA/velocity measurements. Proceedings of the IEEE Aerospace Conference.

[31] Tunyasrirut S. Implementation of a dSPACE Based Digital State Feedback Controller for a Speed Control of Wound Rotor Induction Motor. Proceedings of the IEEE International Conference on Industry Technology.

[32] Frauenholz R.B. (2008). Deep impact navigation system performance. J. Spacecr. Rockets.

[33] Wang F., Cao X.B., Qiu W.X., Zhang S.J., Sun Z.W. (2008). Hardware-in-the-loop validated simulation platform for small satellite control system. J. Harbin Inst. Technol..

[34] dSPACE Inc. (2001). dSPACE Implementation Guide.

[35] Bhaskaran S. Orbit determination performance evaluation of the deep space 1 autonomous navigation system. Proceedings of the AIAA/AAS Space Flight Mechanics Meeting.

